# The Past, Present, and Future of Host Plant Resistance in Cotton: An Australian Perspective

**DOI:** 10.3389/fpls.2022.895877

**Published:** 2022-07-06

**Authors:** Lucy M. Egan, Warwick N. Stiller

**Affiliations:** CSIRO Agriculture and Food, Narrabri, NSW, Australia

**Keywords:** *Gossypium*, host plant resistance, breeding, phenomics, genomics, germplasm utilization, genomic selection

## Abstract

Cotton is a key global fiber crop. However, yield potential is limited by the presence of endemic and introduced pests and diseases. The introduction of host plant resistance (HPR), defined as the purposeful use of resistant crop cultivars to reduce the impact of pests and diseases, has been a key breeding target for the Commonwealth Scientific and Industrial Research Organisation (CSIRO) cotton breeding program. The program has seen success in releasing cultivars resistant to Bacterial blight, Verticillium wilt, Fusarium wilt, and Cotton bunchy top. However, emerging biotic threats such as Black root rot and secondary pests, are becoming more frequent in Australian cotton production systems. The uptake of tools and breeding methods, such as genomic selection, high throughput phenomics, gene editing, and landscape genomics, paired with the continued utilization of sources of resistance from *Gossypium* germplasm, will be critical for the future of cotton breeding. This review celebrates the success of HPR breeding activities in the CSIRO cotton breeding program and maps a pathway for the future in developing resistant cultivars.

## Introduction

Cotton is an important commercial crop grown for its fiber, oil, and protein (Leff et al., 2004; Arora et al., 2017). The cotton genus, *Gossypium*, encompasses approximately 50 species and based on crossing compatibility to the predominant commercial cotton, *Gossypium hirsutum*, the species have been classified into primary, secondary, and tertiary gene pools. The primary gene pool contains five species, while the secondary and tertiary gene pools contain 20 and 25 species, respectively. Globally cultivated cotton utilizes four species; *Gossypium arboreum* (Desi cotton), *Gossypium herbaceum* (Levant or Arabian cotton), *Gossypium barbadense* (Pima, Egyptian, or Sea Island cotton), and *G. hirsutum* (Upland cotton; Wendel et al., 2009; Constable et al., 2015). *Gossypium hirsutum* encompasses 90% of the cotton grown globally, compared to *G. barbadense* (8%), *G. arboreum* (<2%), and *G. herbaceum* (<2%; Constable et al., 2015).

The genetic resources of cotton are spread across five continents and consist of diploid (A–G and K genomes, 2*n* = 2*x* = 26) and tetraploid species (AD genomes, 2*n* = 4*x* = 52) including *G. hirsutum* and *G. barbadense* (Lubbers and Chee, 2009). Munro (1994) stated that the difference in genomes is likely due to geographical isolation. While the C, G, and K genomes are confined to Australia and the D genome to America, the A, B, and E genomes are found across Africa and Asia. The F and G genomes are only found in a single species each. Australia has 17 native *Gossypium* species, containing the C, G, and K genome species (Stiller and Wilson, 2014).

China, India, the United States of America (USA), Pakistan, Uzbekistan, and Brazil are the leading producers of cotton by volume. While Australia only produces around 3% of the world’s cotton, it has the highest yields of any production region and is a significant global exporter (Constable and Bange, 2015). The major cotton-producing states within Australia are Queensland and New South Wales (Kaur et al., 2020), but expansion into the tropical areas of Northern Australia is underway (Yeates, 2003; Yeates et al., 2013). However, the full genetic yield potential of cotton is constrained by both biotic and abiotic stresses which impact yield and production (Constable and Bange, 2015). The intensification of cotton production systems and increasingly variable climate going forwards are encouraging the prevalence of pests and pathogens on cotton. The continued development of cultivars with resistance to target pests and pathogens will aid in decreasing yield losses through crop damage or death. High yielding cultivars contribute to the low cost of production per unit of fiber and ensure profits are sustainable for cotton growers (Constable et al., 2015).

This review aims to highlight the success that cotton breeding activities have had in breeding for host plant resistance (HPR) and looks to future challenges and solutions. The review will build on previous reviews (Wang et al., 2011; Constable et al., 2015; Mubarik et al., 2020; Negm, 2020) and will be largely based on breeding activities in Australian *G. hirsutum*; however, examples and progress in other species will be included. Australia has been particularly successful in deploying genetically modified (GM) cotton cultivars containing insecticidal genes from multinational corporations, like Monsanto, Bayer and Syngenta, targeted at control of key Lepidopteran pests of cotton, such that the entire Australian crop is now GM. This review will, however, only address native or non-GM traits already present within the *Gossypium* gene pool and some novel applications of genetic modification technologies.

## Breeding for Host Plant Resistance

**Figure 1:**
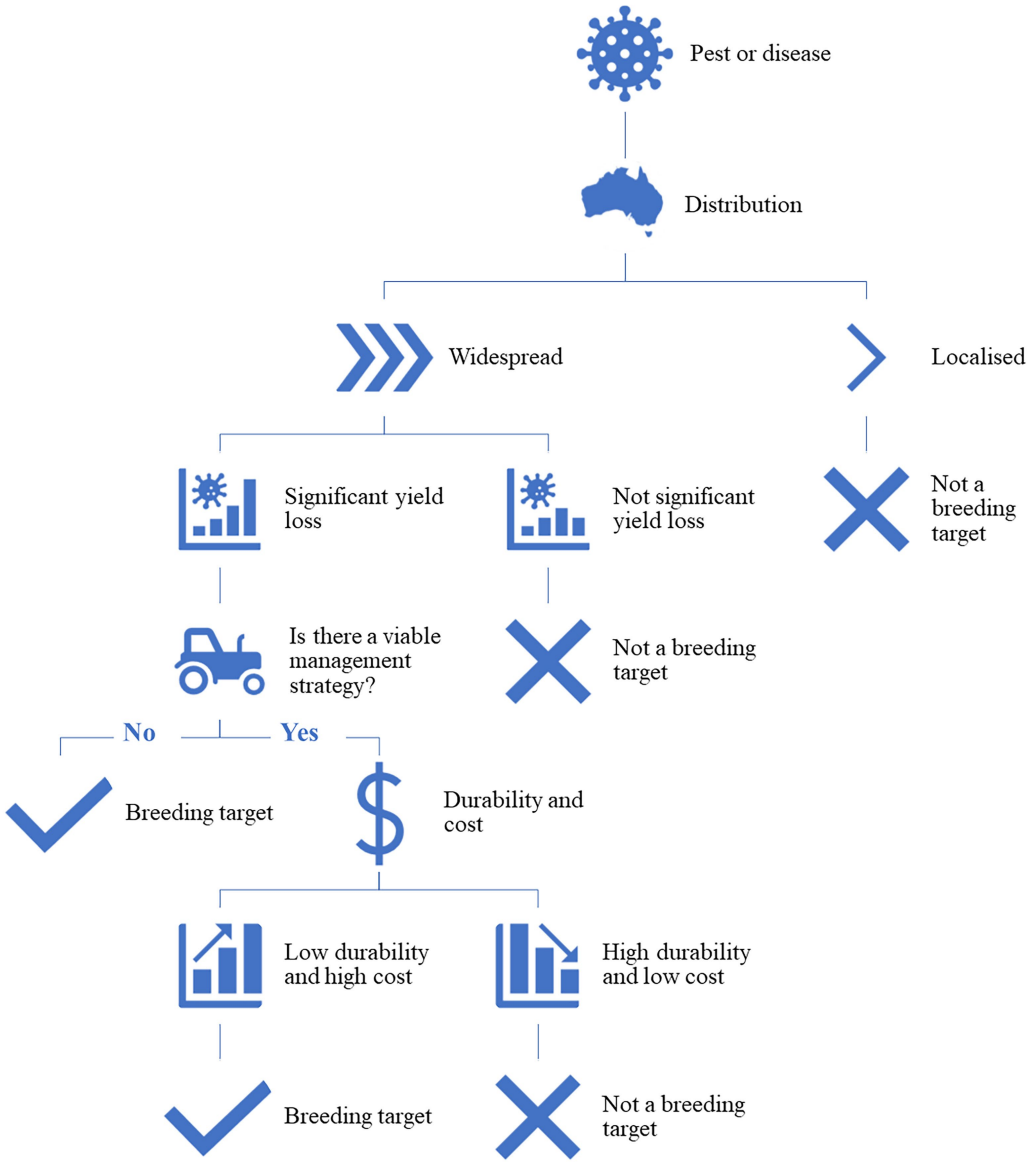
The high-level decision-making process to determine if a breeding program is justified to develop cultivars as a management strategy to control a pest or pathogen.

Host plant resistance is defined as the intentional use of resistant crop cultivars to reduce the negative impacts of pests or diseases on crop production systems (Stout, 2014). Developing HPR cultivars can be the most efficient and effective way for an industry to manage pests and diseases, however, a number of factors need to be considered before any investment in this type of research is committed. Figure 1 outlines a simple decision tree that can be used to justify an investment in HPR research. To start with, the distribution of the pest or disease; if the pest or disease is widespread, causing significant yield loss and there is not a viable agronomic management strategy, then it is a valid breeding target. If there is an appropriate management strategy but it is high cost and has low durability, then HPR is also considered a breeding target. A pest or disease that is very localized or temporally sporadic, has little impact on yield or quality, or is cost effectively controlled by conventional management strategies does not justify the time or cost of HPR breeding. However, the assessment of whether a resistant cultivar is the best method of control of the pest or disease must frequently be revisited as the dynamics of the pest or disease and crop are constantly changing and effective control chemicals can also be unexpectedly lost through changes in government regulations. Breeding for HPR is an on-going process and the adaptation of the pest or pathogen to the developed lines, refinement of the developed lines through further recombination during breeding, and the changing environment from season to season are all variables that need to be considered. There is often a high cost and long timeline associated with developing a resistant cultivar, particularly from the secondary or tertiary gene pools, so to justify the development, return on investment is key.

The standard approach to the development of resistant cultivars can be categorized into three steps: (1) the evaluation of germplasm for possible sources of resistance, (2) introgression of the source of resistance into an elite background, and (3) deployment of the cultivar into existing (or new) crop management systems. The selection criteria used to evaluate germplasm for resistance have to be stringent to ensure that only the most resistant material is carried forward as there are many competing priorities within any breeding program. In the CSIRO cotton breeding program, once the interaction of the crop and pest or pathogen is understood, a wide screen of all available germplasm (we have an extensive germplasm collection of cotton species and cultivars from all around the World) is used to identify lines that show resistance. Where necessary and practical we also import new cotton germplasm into Australia for testing. The screening of these lines involves infecting or infesting the plant with the pathogen or pest and then evaluating survival, damage, or other relevant phenotypic scoring measurements. Disease nurseries, defined as fields that have high levels of the pathogen of interest, are critical for the field validation of resistance. However, the timeframe and high cost of field trials have encouraged the development of glasshouse or growth cabinet bioassays as a more rapid screening methodology, where appropriate. Nevertheless, it is critical that the material is eventually validated in the field. State and Federal biosecurity regulations on-farm limit the potential available area for such field trials as new diseases cannot be purposely introduced for field screening into fields previously lacking a disease.

The difficulty of introducing new resistance into an elite background is governed strongly by the genetic distance of the source of resistance from the cultivated species (i.e., whether it comes from primary, secondary or tertiary germplasm), together with the genetic basis of the resistance and any ploidy differences. Most commonly in cotton, resistance to a pest or pathogen is polygenic, although fortunately some are conferred by single genes. Similar to other polygenic traits, variation in resistance is often continual rather than discrete, and finding germplasm with an appropriate level of resistance can be difficult. The low frequency of occurrence of resistance in elite germplasm, particularly for newly emerging pests or diseases, requires large, wide-scale screens of genotypes to be evaluated. This complexity is confounded if there are environmental interactions between the resistance trait(s) and variability in the pest or pathogen. The introgression of resistance may also be ‘diluted’ if not all of the genes involved are carried across during recombination throughout subsequent generations. As a result, resistance can be partially or completely lost during the breeding process and generations may have to be recovered or recrossed to ensure that effective resistance remains present in introgressed breeding lines. In these situations, the integration of molecular markers into the breeding pipeline is highly beneficial to improve both the speed and the efficiency of the introgression process and can replace a considerable proportion of the phenotypic screening required. If the source of resistance originates from germplasm with a different ploidy to *G. hirsutum*, i.e., a diploid, the first cross may have to involve an inter-specific hybridization and an increase in ploidy and might behave differently to the original diploid, or the resistance may not be viable through the introgression pipeline due to genome incompatibilities.

While establishing a phenotype happens early in the screening process, the development of retrospective tools for breeding, particularly molecular markers, can begin once a source of resistance has been identified and there are many standard procedures for achieving this using modern sequencing technologies (Schneeberger, 2014; Zhu et al., 2017). In plants, it is also common to discover useful pest and disease resistance in wild or unadapted germplasm. Therefore, the integration of resistance into elite populations is often a lengthy process of getting rid of poor alleles brought in from the resistance donor and restoring elite performance to the introgressed progeny. The introduction of essential commercially used GM traits, such as *Cry* genes for resistance to Australian *Helicoverpa* caterpillar species or tolerance to specific herbicides that are the mainstay of our current industry’s pest and weed control strategies, can only commence once a source of resistance has been introgressed into a predominantly *G. hirsutum* genetic background and is stably inherited. Genetic markers linked to all the major genomic regions of the donor that are sufficient to confer resistance are useful to aid in selecting lines with resistance and can decrease the resources needed to produce a resistant cultivar. Molecular approaches have become more feasible over the last decade with low-cost, high throughput sequencing technologies termed “next-generation sequencing.”

### Cotton Breeding in Australia

Cotton breeding began extensively in Australia in the 1960s with the intensification of cotton production systems. Early breeding efforts included pedigree breeding and wide hybrid crosses. Australia’s early breeding programs principally followed programs in the United States, where increased yield and fiber quality traits were at the forefront of breeding targets (Rauf et al., 2019). Cotton breeding in Australia has been dominated by CSIRO and since 2010, we have developed 100% of the commercial cotton cultivars grown here (Kilby et al., 2013). Breeding targets for the Australian industry are primarily focused on yield, fiber quality, disease resistance, and adaptation to all production systems and climatic regions from semi-tropical to cool temperate. Estimated annual genetic gain is reported to be 1–2% *per annum* (Constable et al., 2001; Ward, 2013; Rochester and Constable, 2015) and Constable (2004) stated that for the industry as a whole, breeding has contributed 45% to its progressive yield improvements over the last few decades. Liu et al. (2013) found from an analysis of CSIROs Advanced Line Trials that the increase in yield in Australian cotton cultivars was attributed to genetics (48%), management (28%), and cultivar × management interactions (24%). Conaty and Constable (2020) identified that incorporating HPR into Australian cotton cultivars has been indirectly instrumental in increasing yield, particularly to the pathogens of Bacterial blight (*Xanthomonas citri* subsp. *malvacearum*; BB), Verticillium wilt (*Verticillium dahliae*; VW), and Fusarium wilt (*Fusarium oxysporum* f.sp. *vasinfectum*; FW).

## The Success of Overcoming Biotic Threats to Australian Cotton Production

Australia has a unique combination of clay soils, endemic and introduced pests and pathogens, paired with severe climate extremes, and heavily regulated water use in certain regions. Crop damage caused by pests and diseases is one of the major causes of yield loss (Oerke, 2006). While chemical sprays are the most common form of control (Whalon et al., 2011), large investment and successful efforts to introgress HPR traits into cotton cultivars have occurred (Thomson, 1994; Trapero et al., 2016).

### Bacterial Blight

The first disease to severely impact the Australian cotton industry was BB caused by the pathogen *Xanthomonas citri* pv. *malvacearum* and yield losses of up to 20% were routinely reported (Stiller and Wilson, 2014). In 1985 the okra leaf cultivar Siokra 1–1 was released which had a high level of resistance to BB. Resistance was thought to have originated from the American cultivar Tamcot SP37 carrying multiple blight resistance genes. However, Rungis et al. (2002) suggested that the mapped chromosomal location of the resistance infers that resistance is from a single gene similar to that found in some African cotton cultivars. The development of Siokra 1–1 led to many subsequent BB resistant cultivars which have essentially eliminated losses from the disease in Australia. The resistance is broad-spectrum and provides immunity against all BB strains present in Australia and has remained remarkably stable since it was introduced in 1985. Due to the high uptake (100%) of the BB resistant cultivars, now a compulsory trait for any cultivar released from the CSIRO breeding program, the disease is no longer found in Australian commercial cotton systems (Stiller and Wilson, 2014), although the presence of exotic races of BB overseas means that BB remains a biosecurity risk for our industry.

### Verticillium Wilt

VW is caused by the fungal pathogen *Verticillium dahliae* Kleb and was first reported in Australian cotton crops in the Namoi Valley in 1959 (Evans and Paull, 1967). There are currently three pathotypes of VW present in Australia: two non-defoliating and a defoliating strain (Kirkby et al., 2013). Throughout the 1990s, VW had high incidence levels across the industry with estimated yield losses of 10–62%. In response to this devastation, CSIRO developed and released cultivars including Sicala V-1 (1991) and Sicala V-2 (1994) throughout the 1990s that had a high level of resistance (Stiller and Wilson, 2014). The impact of these cultivars has endured to the current day as much of the resistance found in current commercial cultivars can be traced back to these resistant cultivars.

Over the last two decades, VW has, however, increased in severity and prevalence across the industry (Kirkby et al., 2013) despite the cultivation of VW resistant cultivars and highlights the need for increased levels of resistance in future cultivars. However, the different pathotypes of VW complicate breeding for resistance as they must be treated essentially as separate breeding targets. Recent evidence indicates resistance to one pathotype does not necessarily lead to resistance to another pathotype (Trapero, 2020), so it is assumed that different types of resistance traits will be required. It should be noted that cultivars of cotton resistant to VW strains present overseas are not necessarily resistant to the endemic races, and vice versa.

### Fusarium Wilt

FW is caused by the pathogen *Fusarium oxysporum* f sp. *vasinfectum* and can cause extreme yield loss in some seasons. FW was first identified in Australian cotton systems in 1993 and is now found across the majority of cotton-growing regions (Kochman, 1995). This is a unique Australian race of FW and has proved to be very severe in comparison to other characterized races and biotypes (Wang et al., 2007). The onset of FW in the cotton industry was fast and in response the CSIRO breeding program developed lines with improved resistance within 10 years. Cultivars with high levels of FW resistance including Sicot F-1, Sicala 45, and Sicot 14B were released (Constable, 2006). The impact of these cultivars is reflected in the reports that FW is now not affecting the industry at large, and the rate of spread has slowed significantly in response to the use of resistant cultivars and greater emphasis on on-farm hygiene (Kirkby et al., 2013).

The genetic basis of FW has been a key area of research for the Australian program in the development of molecular markers. Lopez-Lavalle et al. (2012) were the first group to identify quantitative trait loci (QTL) associated with FW in Australia, while earlier studies focused largely on gene expression characteristics of susceptible and resistant cultivars (Dowd et al., 2004; McFadden et al., 2006). However, the genetic basis for resistance is still largely unknown, and the CSIRO program has relied primarily on field phenotyping to date.

### Cotton Bunchy Top

**Figure 2:**
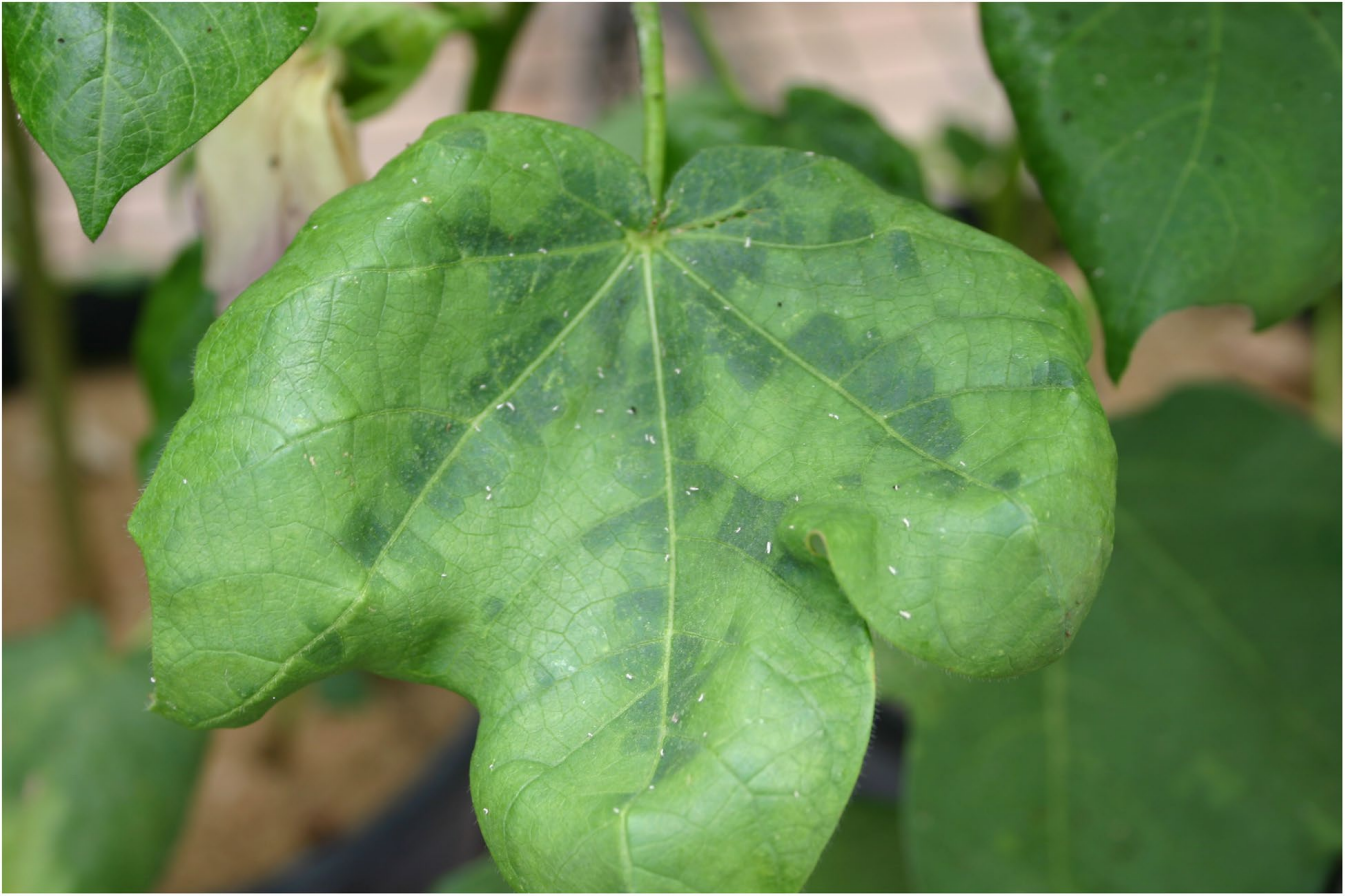
Typical leaf symptoms of a plant with Cotton Bunchy Top (CBT; Photo: Warwick Stiller).

Cotton bunchy top (CBT) is a viral disease that is vectored by cotton aphids (*Aphis gossypii*; Figure 2). It is caused by a *Polerovirus* that appears to be unique to Australia (Reddall et al., 2004; Ellis et al., 2016; Sharman et al., 2021). The disease was first identified in Australia in the late 1990s and nearly all Australian cultivars were susceptible to the disease. However, two cultivars were found that were highly resistant and these are the source being used to breed resistant cultivars. Although widespread, the occurrence of the disease is sporadic and it has rarely occurred at economic levels in the last decade, so the imperative for releasing resistant cultivars is not high. Ellis et al. (2016) identified markers that flanked the single CBT resistance region from the resistant cultivars, and these are now being routinely utilized in the breeding program through marker-assisted selection. The long-term aim is for all our cultivars to be resistant to CBT. The first CBT resistant conventional cultivar, Sicot 620, was released in 2018 and others carrying GM insect and herbicide resistance traits are in the pipeline.

### Morphological Characteristics for Pest Resistance

A number of morphological characteristics have been utilized in Australia to enhance pest resistance in cotton, including the okra leaf shape (deeply invaginated leaf lobes) and reduced or eliminated leaf trichomes (glabrous). Okra leaf cultivars have a more open canopy which results in a less favorable environment for various insect and mite pests (Thomson et al., 1987; Fitt et al., 1992; Wilson, 1994). The report by Butter and Vir (1989) was one of the first to identify the positive correlation between hair density and leaf thickness, and the population of whitefly (*Bemisia tabaci* Genn.). The use of okra and glabrous leaf cotton cultivars has been suggested to minimize the development of Silverleaf Whitefly in cotton crops (Ma et al., 1996; Chu et al., 2002; Miyazaki et al., 2013a).

### Two-Spotted Spider Mites

Secondary pests, such as the two-spotted spider mite (TSSM; *Tetranychus urticae*), have increased in cotton production systems since the introduction of GM traits to control *Helicoverpa* spp. The cultivars with added *Bacillus thuringiensis* (*Bt*) genes have substantially reduced chemical pesticide usage but have allowed several other pests to increase in prevalence. However, the robust resistance that is provided by GM technology has allowed the breeding program to focus on these other pests, particularly sucking pests. Trapero et al. (2016) reviewed the effect on cotton of pests not controlled by GM traits and the available sources of resistance and traits available for HPR.

**Figure 3:**
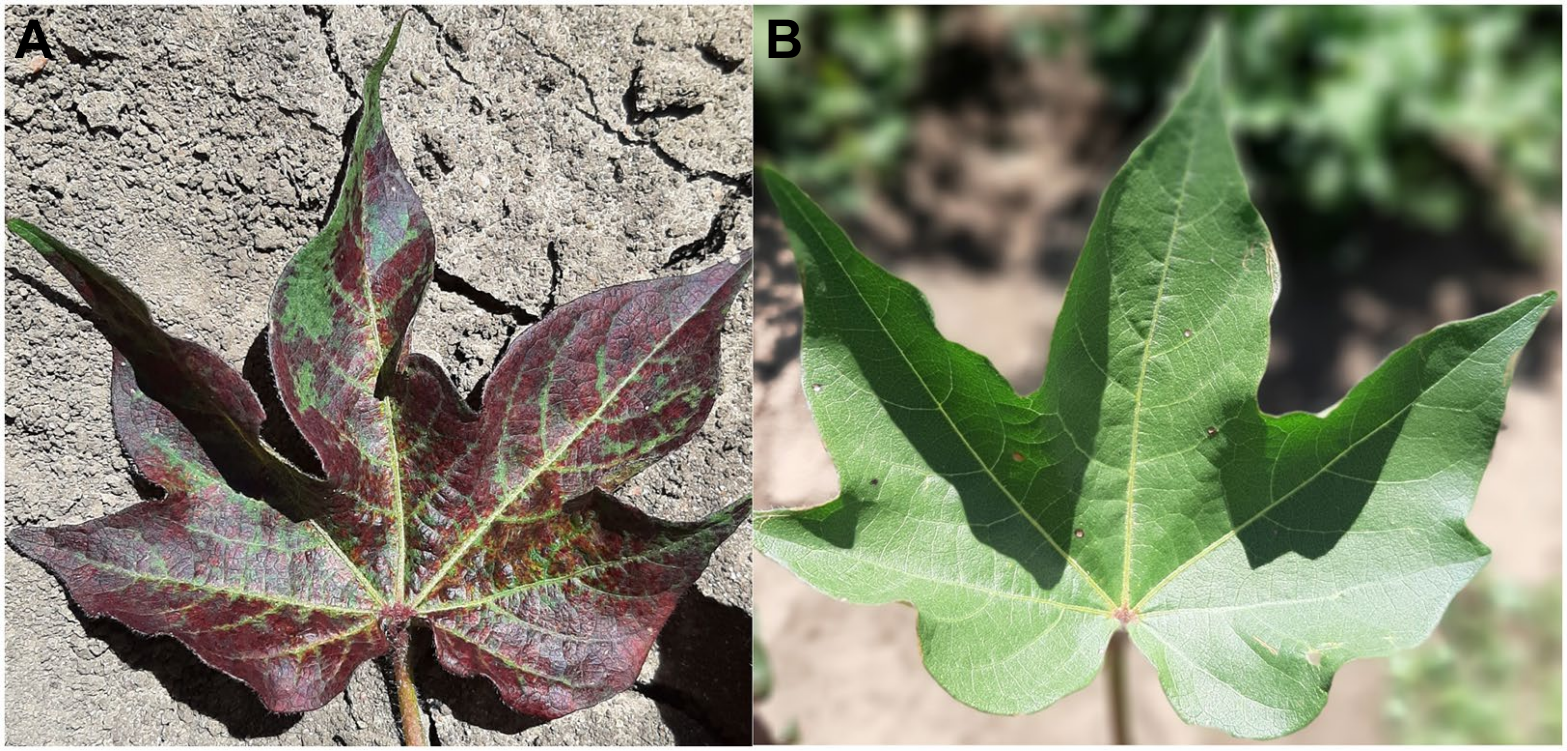
Susceptible **(A)** and resistant **(B)** two-spotted spider mite (TSSM) cotton germplasm from the CSIRO cotton breeding program after infestation with TSSM (Photos: Lucy Egan).

While there are currently no commercial lines with TSSM resistance, resistant germplasm has been identified (Miyazaki et al., 2012; Figure 3). The fitness of two-spotted spider mites was affected more by constitutive than induced resistance traits in those resistant cottons (Miyazaki et al., 2013b), and Miyazaki et al. (2014) found that the jasmonic acid defense pathway is associated with resistance to TSSM in *G. arboreum*.

The past successes of incorporating HPR into the CSIRO cotton breeding program illustrates how the program has identified and deployed effective breeding strategies, which can be carried forward to address future biotic threats to the industry.

### Factors That Have Influenced the Success of the Australian Cotton Breeding Program

Three key factors have played a critical role in why the Australian cotton breeding program has been so successful. Firstly, the program has always had a long-term focus to develop cultivars that meet the needs of the industry. The shared vision between CSIRO and the cotton industry has forged strong partnerships that have informed our breeding targets. The collaboration with commercial partners, Cotton Seed Distributors Ltd. (CSD), Monsanto, and Bayer CropScience, has allowed the continual funding of research over decadal timeframes and has allowed the marketing of our cotton cultivars both nationally and globally under exclusive License arrangements through CSD. Although the CSIRO cotton program is nominally a public breeding program, the primary focus is cultivar development, and the research institution allows a great depth of interdisciplinary research to occur to ensure we are at the forefront of modern breeding technologies. Economic assessments of CSIRO’s cotton breeding program show an estimated 80:1 return on investment (Centre for International Economics, 2002; ACIL Allen Consulting, 2014), justifying an on-going investment. Secondly, the Australian climate, environment and soil types provide a conducive selection environment to make genuine genetic gain. The development and refinement of breeding strategies over a long period have allowed the program to take full advantage of the selection environments across a range of testing locations. In addition to the selection environment, the breeding and selection strategies are fine-tuned to current management systems to exploit the highest rate of genetic gain. The high level of crop management that the industry uses is a critical contributor. Thirdly, grower co-operation to provide sites for field experiments is critical so that the breeding material can be fully tested before commercialization. In particular, the volunteering of diseased fields by growers is the most important aspect of disease resistance breeding so that the material can provide a reliable and industry relevant phenotype (Ward, 2013).

## Emerging Biotic Threats to Australian Cotton Production

While the Australian cotton breeding program has been successful in developing cultivars that have a high level of resistance to target pests and pathogens, the level of resistance is constantly monitored as it may be overcome as the pathogens or pests evolve, or the resistance level may inadvertently decline if not constantly selected for. The anticipation of new pests and diseases that could enter Australian cotton production systems is a constant threat and is monitored closely across the country by biosecurity teams and is used to inform the CSIRO breeding program of possible new breeding targets. The strict quarantine procedures for the importation of materials that may contain new pests and diseases into Australia reduces biological threats. However, research activities that could be beneficial to combat potential new pests and diseases are greatly restricted. Cotton Leaf Curl (*Begomovirus*), for example, is a viral disease vectored by B-type Whiteflies and is widely endemic across parts of Asia with devastating effect. The insect vector, but not the virus, has managed to find its way to Australia, but it is difficult for us to evaluate or breed for material that may be resistant to the virus as it would be foolhardy and illegal to bring the virus into this country. Nevertheless, we can bring some purportedly resistant germplasm into Australia to have some material here should a viral incursion ever occur. Examples of emerging endemic threats include Black root rot (BRR) and secondary pests arising from the decline in broad spectrum pesticide usage on Australian cotton cultivars containing GM traits.

BRR is caused by the pathogen *Berkeleyomyces rouxiae* sp. nov. (previously *Thielaviopsis basicola*) and was first reported on cotton in Australia in 1990 (Allen, 1990) and is now present in all cotton-producing areas of Australia (Nehl et al., 2004). BRR severity and incidence is controlled largely by the climate; cooler and wetter conditions exacerbate symptoms. Kirkby et al. (2013) stated that long-term survey data have shown the exponential growth and dispersal of BRR over decades, particularly into cooler southern regions of New South Wales where the industry has expanded in the last decade. Plants with BRR show stunted growth early in the season, delayed flowering, blackening of the roots and a reduction in the number of lateral roots (Mims et al., 2000; Allen, 2001; Pereg, 2013). Allen (2001) noted that sources of resistance to BRR in Australian *G. hirsutum* or *G. barbadense* germplasm were lacking for traditional breeding purposes. Although advances have been made in utilizing genetic modification for resistance to BRR using pathogen defense genes from other species (OGTR, 2006), toxic effects and undesirable phenotypic characteristics have been reported in the GM plants (Pereg, 2013) and they have not been progressed to commercial use. As global breeding efforts have also yet to be successful in developing a BRR resistant commercial cultivar., disease management is currently based on cropping practices. However, BRR remains a breeding target in the CSIRO program and Wilson et al. (2021) characterized and mapped strong resistance to BRR to a single region from *G. arboreum* that contains several putative resistance-like genes.

## Future Directions

The future of global cotton production is reliant on the native variation available in cotton germplasm and potentially novel sources of new GM traits to develop high-performing cultivars. The available variation to exploit for future traits has to be either present in germplasm or created through recombination with related species or through transgenic techniques. An understanding of the history of Australian cotton breeding and how breeding successes were achieved to date is paramount to attaining and sustaining future genetic gain. The success of utilizing traditional breeding methods has been apparent in Australian cotton breeding activities, and if similar challenges are encountered, the industry should feel confident that our breeders can utilize a similar methodology for success in the future and the timeframes involved. Quantitative breeding methods including genome-to-phenome knowledge, statistics, genomic selection (GS), and fundamental quantitative genetics approaches will all advance the understanding and utilization of the natural variation in cotton germplasm (Cooper et al., 2014) and hopefully speed up the process. The recent advancements in panomics indicate that these new breeding technologies should continue to be integrated into HPR cotton breeding programs alongside existing and new crop management strategies and the continual search for new sources of resistance.

### Crop Management

While breeding for HPR will be critical for future climates, the role of crop management and precision agriculture should not be underestimated and should be considered if it is determined that a breeding solution is not the most appropriate path to success (Figure 1). For example, precision agriculture techniques can allow farmers to have a detailed understanding of where a disease may occur within a field, or across a farm, and consequently implement more efficient management strategies specific to their situation. Techniques can range from utilizing high level geospatial data through to complex models of precision application of insecticides and fertilizers to reduce the impact of plant pests and diseases on the crop (Shafi et al., 2019; Roberts et al., 2021). New pesticides and fungicides are under continuous development by industry and new approaches to control such as using exogenously applied RNA interference (RNAi) molecules that specifically inhibit growth or development of pests or pathogens remain as viable alternatives to HPR if they can overcome some of the technical and economic hurdles to large scale production (Hernández-Soto and Chacón-Cerdas, 2021).

### The Identification of New Sources of Resistance

The continued identification of new sources of resistance and an ability to reliably screen for that resistance at scale will be the most critical activity for future HPR breeding activities. If resistance cannot be identified or screened for, it cannot be incorporated into the cultivar development pipeline. To incorporate an accurate and effective laboratory, growth cabinet or glasshouse screening tool into the HPR breeding pipeline, the method will need to be developed and optimized in parallel once individual sources of effective resistance are first identified. Screening methods should be fast, cheap, repeatable, and the results are obtained reflective of the field situation to provide the greatest value to a breeding program.

In the absence of an accurate high throughput screening method, it is difficult to identify resistance and the rate of genetic gain will be slowed. The time to develop a screening method varies greatly and is dependent on the pest or pathogen. However, already published methods in the literature can often be used and adapted. For example, within 12 months, the CSIRO cotton breeding program developed a controlled environment rapid seedling assay to screen for TSSM resistance. The method was optimized over the following two years until a final protocol was developed (Trapero, 2022b). Similarly, a controlled environment seedling VW screening assay was developed within 12 months (Trapero, 2022a). However, although the VW bioassay produces reproduceable data, the high environmental interactions of this pathogen result in the assay being only partially predictive of how some genotypes respond to VW under field conditions. Genotypes with susceptibility or low levels of resistance can be identified and eliminated, but there is not good discrimination among moderate or highly resistant genotypes. Field-based, season long evaluation trials should always be used to validate any lab or controlled environment screening methods, particularly for complex pests and pathogens.

### The Impact of New Breeding Technologies

Introgression of target traits from related species by traditional breeding is a lengthy process, often taking more than 20 years to reach a viable commercial outcome. In comparison, introgressing traits from elite cotton and non-elite primary gene pool germplasm takes approximately 10 and 15 years, respectively. The incorporation of new breeding technologies promises to allow better incorporation of sources of resistance from outside of elite germplasm and the stacking of resistance genes.

**Figure 4:**
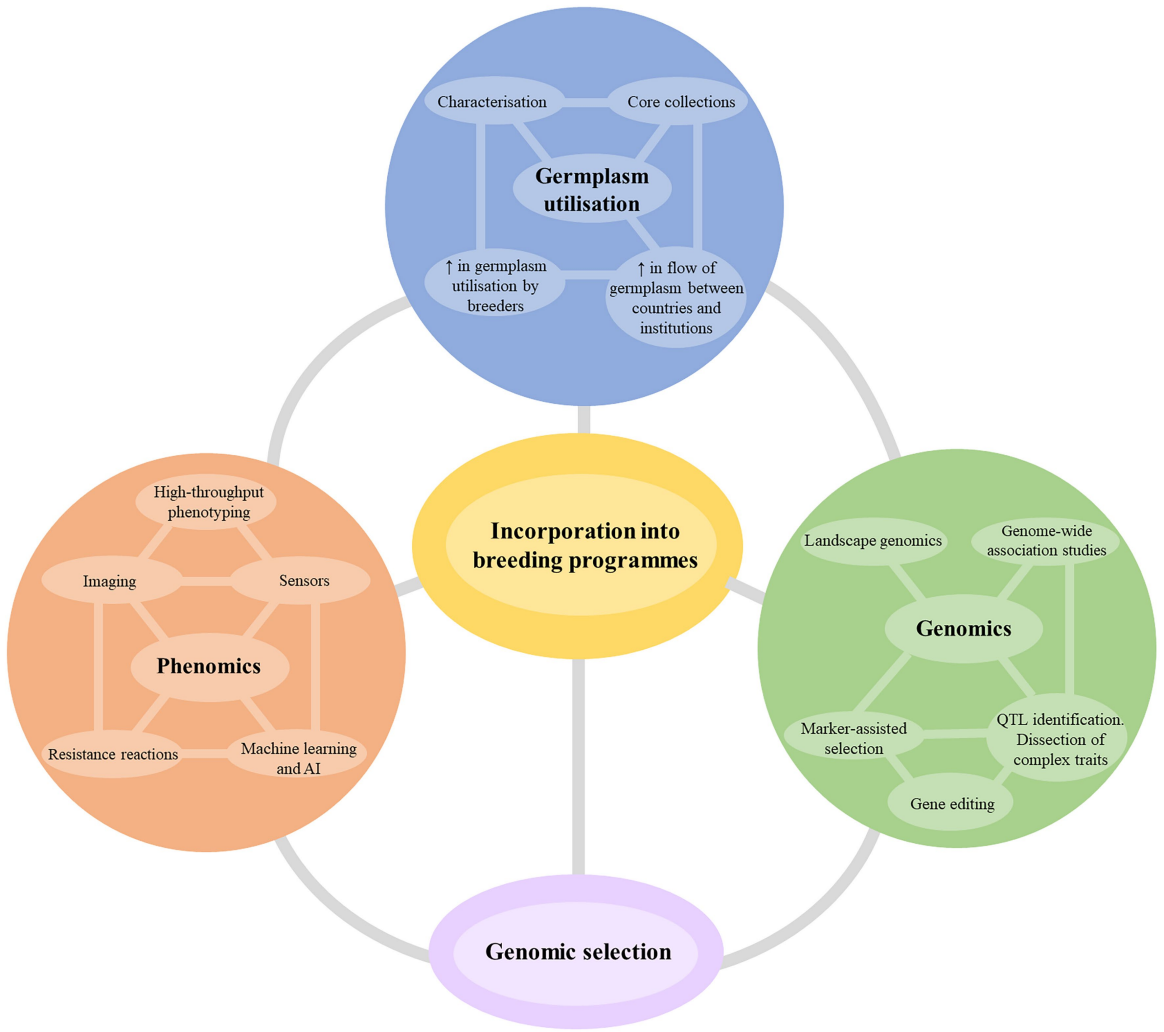
The potential future strategies for improvement of host plant resistance in cotton breeding programs. AI, artificial intelligence; QTL, quantitative trait loci.

New breeding technologies that increase the accuracy and speed of selection are a significant area of investment for breeding research. Although the technologies may not always accelerate the breeding timeline significantly (often only by 1–2 years), they facilitate superior line selection at greater accuracy and increase the overall rate of genetic gain over time. Figure 4 summarizes the suggested themes and breeding technologies that any cotton breeding program should target to develop cultivars for future challenges. While it is impractical to expect a silver bullet from one technology or methodology, the most effective strategy will be the incorporation of several technologies depending on the nature and complexity of the resistance traits being incorporated.

#### Phenomics

The field of phenomics (the measurement of sets of phenotypes, often physical or biochemical traits) offers huge potential to decrease the current high cost of field phenotyping of plant resistance responses and is becoming increasingly incorporated into commercial HPR breeding programs. One of the main benefits of phenomics is that the classification of resistance reactions is often non-invasive and standardized across fields, reducing human error (Mahlein et al., 2019). Digital phenotyping, defined as the use of sensors (e.g., infrared sensors), robotics, imaging, machine learning and artificial intelligence, has been a focus of recent research to characterize, quantify, and monitor plant-pathogen interactions (Mutka and Bart, 2015; Singh et al., 2016; Mahlein et al., 2019; Li et al., 2020a). Of the technologies available, sensors and various spectral (multispectral and hyperspectral) imaging appear to be the most advanced and have the technical and functional capability to be incorporated into breeding programs (Kuska et al., 2015; Thorp et al., 2015). Sensors have been widely used to monitor resistance reactions during plant defense mechanisms (Singh et al., 2016; Mahlein et al., 2019), albeit none have been published in cotton.

Several obstacles, largely based on the size and type of data, still need to be overcome for phenomics technologies to be widely adopted in cotton breeding programs. First, it is critical that datasets utilized for phenomic technologies are large and can be utilized by several software programs without requiring complex data transformations or re-formatting. Second, the technologies need to be scalable and useable in the field with current management practices. High-throughput phenotyping methods have begun to overcome some of the originally proposed challenges, such as automation, accuracy, and upscaling. However, applicability across crops and diseases/pests remains a challenge (Varshney et al., 2021). Third, the data collected should be multi-trait and multi-environment to increase prediction accuracy as diseases and pests can cause multiple pleiotropic symptoms and be influenced by environmental conditions over the life of the crop. The incorporation of environmental data will be integral in moving forward with phenomic tools for pest or disease phenotyping. The incorporation of genetic technologies with phenomics should begin to unravel the genetic interactions with the pests or pathogens and the environment and could enhance the breeding process by highlighting what environmental factors are important for specific target traits. Finally, data processing techniques and capacity will need to increase to ensure the accuracy and robustness of predictions and to cope with the significantly larger volumes of data that would be generated in any commercial breeding enterprise. Although the development of data processing techniques for machine learning and artificial intelligence (AI) tools are largely in the developmental stage (Mahlein et al., 2019), the use of AI to facilitate the phenomics process will provide high levels of effectiveness and efficiency to develop solutions (Li, 2020). In an HPR scenario, the AI could identify what traits are important in HPR and machine learning could find the correlations, trends, and environmental parameters that are important for performance. However, like any phenotyping process, unless the data are collected accurately, clear decisions cannot be made.

Further characterization and dissemination of the relationship between the plant, the pathogen/pest, and the farming systems in which they are found are needed. This responsibility will fall to research teams, where a large cohort of deep knowledge needs to be shared in order to deploy phenomics tools into breeding programs. Particularly, plant breeders will need to be open and proactive at realizing the potential that phenomics tools could have to advance their breeding programs (Giglioti et al., 2015; Cobb et al., 2019). However, it is important to remember that phenomics will not always shorten the timeline for cultivar development; rather, it will facilitate an upscale and increase in accuracy of phenotyping capability.

#### Genomics

The field of genomics has seen considerable advancement in recent decades mostly due to the rapidly decreasing costs of genome sequencing and democratization of sequence analysis tools out of the hands of specialist bioinformaticians (Mansoor and Paterson, 2012; Nadeem et al., 2018; Zaidi et al., 2018). In a report by the National Academies of Sciences and Medicine (2019), genomics and precision breeding were identified as the two areas that will have the largest impact on food and agricultural production in the coming decades. Genomic breeding methods aim to enhance the speed of breeding through the exploitation of minor effect alleles, reducing linkage drag, and increasing the accumulation of favorable alleles to sustain and increase the genetic gain (Varshney et al., 2021). The advances in short and long read sequencing technologies have enabled many more plant genomes to be sequenced, and the sequencing of diverse germplasm within cotton species has identified many candidate genes that could be exploited for future traits.

##### Genomic Selection

The concept of GS was first proposed by Meuwissen et al. (2001) and takes into account the small effects of all of the genetic markers available across a genome to determine an estimated breeding value for any individual based on its particular set of marker combinations through a robust statistical model developed from a large training population of individuals or lines of known genotype and phenotype for the traits of interest. Many studies have looked at the use of such whole-genome prediction models and GS approaches for disease resistance traits in wheat (Ornella et al., 2012; Rutkoski et al., 2012, 2014; Arruda et al., 2015), maize (Technow et al., 2013), cassava (Ly et al., 2013), potato (Enciso-Rodriguez et al., 2018), and barley (Lorenz et al., 2012; Sallam et al., 2015; Tiede and Smith, 2018), but there are currently yet to be any published in cotton.

As the genetic complexity of resistance shifts from a single or few major R genes, to pyramiding multiple minor QTL, marker-assisted selection for disease traits will likely be replaced by GS (Poland and Rutkoski, 2016). A GS model has been proposed as useful for HPR that is controlled by a combination of a few large-effect QTLs in a polygenic background (Rutkoski et al., 2011). However, the deployment of GS into a breeding program requires a reliable phenotype and large amounts of phenotype data for the target environment across a sufficiently large training population to ensure accuracy in the model developed (Poland and Rutkoski, 2016). Exploiting a selection index could be an opportunity in cotton breeding once a robust trained GS model is built. However, large amounts of environmental data would need to be included in the model, for many of our target diseases whose incidence and symptoms are regulated by climatic factors.

The plant-pathogen and environment interactions heavily influence the variability of the phenotype data, particularly in disease field trials where there is an uneven distribution of the disease in the field and considerable year to year variation in the climate. Therefore, the incorporation of environmental data in genomic predictions allows for more stability of genotype selection across different growing sites, regions, and years. A useful tool in this area may be landscape genomics. This is a developing research field that aims to identify the relationship that exists between adaptive genetic imprints on the genome and environmental heterogeneity (Joost et al., 2007). The technique can identify genetic variants that have contributed to local adaptation (Manel et al., 2010) but require many markers to cover the whole genome. Although landscape genomics studies have been conducted for over a decade, basic theoretical frameworks are still minimal (Li et al., 2017). To date, there are no published studies in cotton. Landscape genomics could be utilized in HPR studies, whereby the host plants will exhibit different allele frequencies between resistant and susceptible plants. The approach could be further utilized by correlating genomic regions with environmental characteristics and could aid in explaining how the change in an environmental characteristic could affect resistance (Parisod and Holderegger, 2012; Rellstab et al., 2015). Landscape genomics could be utilized by a GS model as it will take into account what alleles are required for the expression of resistance in specific environments.

More frequently, Genome-Wide Association Studies (GWAS) are also being incorporated into genomic selection models to leverage the genotype and environment interaction data. While GWAS’s have been successful in cotton in detecting QTL for fiber quality traits and yield components (Su et al., 2016; Fang et al., 2017; Gapare et al., 2017; Liu et al., 2018; Li et al., 2020b), and several studies have identified candidate genes for Verticillium wilt resistance (Li et al., 2017; Gong et al., 2018; Abdelraheem et al., 2020; Zhang et al., 2020), there is now the opportunity to leverage that data and incorporate it into a GS model (Moore et al., 2019). Identifying the main environmental trends from GWAS’s could help in predicting the performance of complex traits, such as HPR, through GS (Li et al., 2021).

Often GWAS and GS models have been used independently, but peak associated markers have been incorporated into GS models as a fixed-effect covariate (Zhang et al., 2014; Arruda et al., 2016; Spindel et al., 2016; Rice and Lipka, 2019). Previous studies imply that utilizing both methods could aid in predicting traits controlled by many small-effect QTLs, such as HPR. However, increased efforts towards fine mapping of genomic regions controlling biotic resistance are needed (Shen et al., 2010; Zhao et al., 2017).

Gene-based breeding (GBB) technologies are an alternative method to GS. GBB differs from GS whereby technology is used to find genes that are involved in a process, i.e., resistance to a pest or disease. If there are several hundred genes that are known to be involved in a pathway or process, usually identified through transcriptomics or GWAS, algorithms can be created based on the contribution of those genes. As GBB only looks at a subset of genes, compared to GS that scans the whole genome, GBB may give a better outcome than GS. However, GBB is best utilized when targeting a single or limited number of traits. If several traits are being selected, GS would be more beneficial as the whole genome would be covered. However, the GS and GBB technologies are not mutually exclusive and should be used together to increase impact and improvement for breeding efforts. GBB could be utilized in early HPR breeding efforts whereby germplasm is selected primarily based on the level of HPR. Once the resistance is incorporated into an elite background, GS could be used to maintain the resistance in future cultivars.

##### Gene Editing

Gene editing (GE) in plants refers to the manipulation of genetic material by inserting, deleting, or replacing DNA sequences using sequence targeted nucleases. GE can convert alleles to a specific sequence in a different background and has the potential to generate resistance in an elite line in as little as one generation in some amenable plant species. GE also has the potential to create novel directed variation that could be utilized to address future breeding objectives (Nasti and Voytas, 2021). GE often results in fewer unintended genomic changes compared to earlier random mutagenesis techniques (Graham et al., 2020) and is more precise than those methods being able to specifically alter a native gene of a crop (Jung et al., 2018). This technology could allow the identification of target genes that are involved in resistance and for them to be incorporated into an elite background or could convert susceptible alleles to resistant alleles in an elite background.

The CRISPR/Cas RNA-guided system is the most widely used form of GE (Khan et al., 2018) and has been highly effective for site-specific genome engineering (Peng et al., 2020; Zaidi et al., 2020). More recently, CRISPR has been used to activate (CRISPRa) or suppress (CRISPRi) genes. The use of CRISPR/Cas nickases for promoter manipulation could be helpful in the regulation of gene expression and reduce the associated risks of introducing foreign material into the genome.

Gene editing technologies, in partnership with enhanced knowledge of trait architecture, will enable possible solutions to engineer complex trait variation (Varshney et al., 2021). The variation created through GE can be beneficial when there is no current trait of interest in the available germplasm (Holme et al., 2019). A prime candidate for GE could be BRR resistance. Currently, there is no known resistance in any tetraploid cotton cultivars in the world, although we have identified good resistance in the diploid *G. arboreum*. If the underlying resistance gene(s) can be identified and related but ineffective R genes are present in tetraploid cotton, targeted mutagenesis could potentially convert those genes and generate resistant candidates in a much shorter timeframe than interspecific introgression that often takes more than 20 years. GE can be used to target resistance genes, silence susceptibility genes, and stack multiple R genes together for robust and resilient resistance. Li et al. (2022), for example, used precision GE to delete a small segment of the wheat powdery mildew (*Blumeria graminis* f. sp. *tritici*) susceptibility genes, MLO, present in the B sub-genome of hexaploid wheat to produce a cultivar resistant to the fungal pathogen in a much shorter time than could be achieved by backcrossing the known mutant of that gene. The pyramiding of target resistance genes to reduce the breakdown of resistance to target pests and diseases will be a key area of interest for future GE activities in cotton and other crops. However, the challenge in cotton is that the plants and pathogens/pests do not often have simple gene for gene resistance mechanisms (except for BB), and resistance is often broad-spectrum conferred by multiple genes. GE may also be used to target DNA viruses by expressing the editing system in the host plant nucleus to target DNA viruses when they enter and begin to replicate [reviewed in Borrelli et al. (2018)].

Although there are many benefits of using GE, a large constraint in the commercial context is the legal licensing and regulatory obligations that it entails as well as the public perceptions of GE. The patent landscape for GE technologies is quite complex and obtaining the rights to use GE for a commercial purpose can be a minefield. Also, in Australia, at least, taking a whole resistance gene out of a diploid species, say, and inserting it into a commercial cultivar or any deliberate changes to the sequence of a gene using a directed repair of a break within the gene to convert it to a resistant allele, is currently defined as genetic modification and subject to very expensive and time-consuming regulatory requirements. However, as the plant breeding industry globally moves more toward using GE technologies in many crops and horticultural plants it may not always be considered GM as it will become more familiar and less novel. Recombination-based breeding, or traditional breeding, is the way that cotton breeding will continue while the State or Federal governments, and/or public policy limit the use of these new breeding technologies in agriculture. Critically, public perceptions of what GE technologies and GM cultivars are, will be key and will inform any future changes to the legal definitions. Cotton growers in Australia already accept genetic modification techniques and have seen the value they bring to the industry, so would likely get behind any new cultivars developed using GE.

##### RNA Interference

The use of RNAi is a major possibility in future cotton applications as it is a crop that is now firmly based on the use of GM traits for crop protection and weed control. RNAi is a post-transcriptional process that results in the suppression of gene expression through the degradation of the target mRNA (Fire et al., 1998). The RNAi molecule can be delivered either through the plant as a “transgene” or applied ectopically like a chemical pesticide or fungicide. Investments in research on exogenously applied RNAi has increased in recent years, largely due to the significant reductions in cost of production of RNA that need to get to the point of being similar in cost to synthetic chemicals. GM RNAi crop cultivars have already been developed and commercialized in corn, where the plant produces in its roots a double-stranded RNAi molecule targeted at a gene in the larvae of the corn root worm *Diabrotica virgifera virgifera* LeConte and delivered when the insect eats the roots [reviewed in Darlington et al. (2022)]. The understanding of how resistance genes work and how they recognize pathogens could lead to the development of “purpose-designed resistance genes” that will target specific factors in pathogens and could become a powerful tool in the HPR arsenal. Plants could express multiple RNAi molecules that target a range of pest and pathogen genomes, but application of RNAi is currently limited by the identification of effective gene targets in the pests or pathogens. Synthetic biology holds promise to overcome the target identification limitation and the continuation of sequencing efforts for pests and pathogen genomes will encourage the use of RNAi technology in future control strategies, particularly through exogenously applied RNA as it has a much simpler and shorter regulatory timeframe when compared to GM crops. Once effective molecules and gene targets are identified with exogenous RNAi the genetic modification approach may become more amenable.

#### Germplasm Utilization

Increased germplasm utilization from *Gossypium* species to identify new HPR traits is strongly encouraged. Genebanks hold a range of species and diversity, and utilizing these is the cheapest and most efficient way to futureproof cotton and improve progress in genetic gain (Liu et al., 2013; Egan et al., 2019). However, the lack of characterization of germplasm in the genebanks deters breeders from utilizing that material. Linkage drag from unimproved germplasm is a well-reported problem and an obstacle that needs addressing to encourage the incorporation of new germplasm crosses into breeding programs (Acquaah, 2009). Although crossing different *Gossypium* species is possible (Brubaker et al., 1999; Bell and Robinson, 2004; Tahir and Noor, 2011), the difficulties in crossing and producing fertile seed, coupled with the frequency of deleterious alleles piggy-backing with the desired trait highlight why very few have made it into commercial cultivars (Ganesh Ram et al., 2008). Ultimately, the minor *Gossypium* species can never compete with commercial cultivars on yield and fiber traits. Deep characterization of germplasm held in global genebanks could be a pathway to overcome the bottleneck when combined with the development of new breeding technologies that may allow more targeted transfer of segments of donor genomes carrying desirable traits and increase the precision of introgression breeding without impacting on agronomic performance.

Finally, the integration of breeding, agronomy and farming systems management, and the techniques of phenomics, genomics, and germplasm utilization are all needed to identify and introduce new sources of HPR into Australian cotton and have them successfully deployed. However, none of the above-mentioned techniques will be the silver bullet for the future of HPR breeding in cotton, or any plant breeding activities. An approach that encompasses and integrates several technologies and field testing across multiple environments still needs to be adopted (Li, 2020).

## Conclusion

As cotton production increases in intensity and scale, so will the number of existing and new cotton pests and diseases. The lessons learnt from the past six decades of HPR breeding activities in the CSIRO cotton breeding program can be used to advise and inform other programs on future activities. Historical breeding efforts show that Australia has been successful in developing high-yielding cultivars with resistance to a range of pests and pathogens, primarily achieved through traditional phenotypic selection techniques and targeted germplasm utilization. However, future cotton breeding efforts face significant challenges in continuing to identify and incorporate HPR.

Genomic breeding technologies have both significant benefits but also challenges. For HPR, this lies in the complexity of resistance often found in cotton and how it will be packaged with commercial insect and herbicide resistance traits in an elite background. Plant breeders need to exploit recombination and stack resistance genes to improve their annual genetic gains. However, this is often not possible through conventional recombination breeding as the population sizes needed grow exponentially with increasing numbers of traits being selected. There will continue to be a trade-off between the breeding aims and the optimal outcome that can be achieved. In the CSIRO cotton breeding program, five GM traits for pest resistance are now incorporated into elite breeding lines as well as several native traits. Currently, the program is investigating GS to address these challenges. However, GE is a possible future option to improve HPR traits and would be of excellent value where several allele changes can be edited in one generation, rather than cycling through several generations and diluting past successes in cultivar improvement.

Succinctly, GS is the way forward for the present and near-future, but there will be some occasions where it will be better to utilize GE so that the elite background is not lost. GE will likely be used in the future to overcome challenges associated with recombination breeding and to allow access to traits confined in the tertiary cotton gene pool, particularly the diploid species. However, this relies on having an efficient, genotype independent transformation system or a transgene-free editing system as the challenge of packaging all of the required traits into an elite genetic background remains and is the ultimate challenge for a breeder.

In conclusion, future efforts and resources in HPR cotton breeding should focus on the development of rapid resistance screening methodologies that are predictive of the field, the continued identification of new sources of resistance both within cultivated cotton and its near relatives, and the adoption and integration of modern phenomic and genomic tools in day-to-day breeding. These methods can be incorporated into traditional phenotypic breeding pipelines to enhance the opportunities to accelerate and sustain the rate of genetic gain. GE can deploy a simple and rapid solution of HPR for genetically simple traits, but for complex HPR traits, GS will need to be used. The use of these genomic technologies will help inform crossing and selection decisions but will not be a substitute for robust field phenotyping and validation.

## Author Contributions

LE conceived and drafted the review. LE and WS designed and wrote the review. All authors contributed to the article and approved the submitted version.

## Funding

This work was supported by Cotton Breeding Australia, a joint venture between Cotton Seed Distributors Ltd. Australia and CSIRO Agriculture and Food. The authors declare that this study received funding from Cotton Seed Distributors Ltd. Australia. The funder was not involved in the study design, collection, analysis, interpretation of data, the writing of this article or the decision to submit it for publication.

## Conflict of Interest

The authors declare that the research was conducted in the absence of any commercial or financial relationships that could be construed as a potential conflict of interest.

## Publisher’s Note

All claims expressed in this article are solely those of the authors and do not necessarily represent those of their affiliated organizations, or those of the publisher, the editors and the reviewers. Any product that may be evaluated in this article, or claim that may be made by its manufacturer, is not guaranteed or endorsed by the publisher.

## Abbreviations

AI: Artificial intelligence
BB: Bacterial blight
BRR: Black root rot
CSIRO: Commonwealth Scientific and Industrial Research Organisation
CBT: Cotton bunchy top
CSD: Cotton Seed Distributors
FW: Fusarium wilt
GBB: Gene-based breeding
GE: Gene editing
GM: Genetically modified
GWAS: Genome-wide association studies
GS: Genomic selection
HPR: Host plant resistance
QTL: Quantitative trait loci
RNAi: RNA interference
TSSM: Two-spotted spider mites
USA: United States of America
VW: Verticillium wilt
